# PI3K p110δ Is Expressed by gp38^−^CD31^+^ and gp38^+^CD31^+^ Spleen Stromal Cells and Regulates Their CCL19, CCL21, and LTβR mRNA Levels

**DOI:** 10.1371/journal.pone.0072960

**Published:** 2013-08-29

**Authors:** Teresa M. Zotes, Roberto Spada, Vladimir Mulens, Sonia Pérez-Yagüe, Carlos O. Sorzano, Klaus Okkenhaug, Ana C. Carrera, Domingo F. Barber

**Affiliations:** 1 Department of Immunology and Oncology, Centro Nacional de Biotecnología-Consejo Superior de Investigaciones Científicas, Madrid, Spain; 2 Biocomputing Unit, Centro Nacional de Biotecnología- Consejo Superior de Investigaciones Científicas, Madrid, Spain; 3 Laboratory of Lymphocyte Signalling and Development, Babraham Institute, Cambridge, United Kingdom; McGill University, Canada

## Abstract

The role of p110δ PI3K in lymphoid cells has been studied extensively, showing its importance in immune cell differentiation, activation and development. Altered T cell localization in p110δ-deficient mouse spleen suggested a role for p110δ in non-hematopoietic stromal cells, which maintain hematopoietic cell segregation. We tested this hypothesis using p110δ^WT/WT^ mouse bone marrow to reconstitute lethally irradiated p110δ^WT/WT^ or p110δ^D910A/D910A^ (which express catalytically inactive p110δ) recipients, and studied localization, number and percentage of hematopoietic cell subsets in spleen and lymph nodes, in homeostatic conditions and after antigen stimulation. These analyses showed diffuse T cell areas in p110δ^D910A/D910A^ and in reconstituted p110δ^D910A/D910A^ mice in homeostatic conditions. In these mice, spleen CD4^+^ and CD8^+^ T cell numbers did not increase in response to antigen, suggesting that a p110δ^D910A/D910A^ stroma defect impedes correct T cell response. FACS analysis of spleen stromal cell populations showed a decrease in the percentage of gp38^−^CD31^+^ cells in p110δ^D910A/D910A^ mice. qRT-PCR studies detected p110δ mRNA expression in p110δ^WT/WT^ spleen gp38^−^CD31^+^ and gp38^+^CD31^+^ subsets, which was reduced in p110δ^D910A/D910A^ spleen. Lack of p110δ activity in these cell populations correlated with lower LTβR, CCL19 and CCL21 mRNA levels; these molecules participate in T cell localization to specific spleen areas. Our results could explain the lower T cell numbers and more diffuse T cell areas found in p110δ^D910A/D910A^ mouse spleen, as well as the lower T cell expansion after antigen stimulation in p110δ^D910A/D910A^ compared with p110δ^WT/WT^ mice.

## Introduction

Secondary lymphoid organs (SLO) are sites of highly organized lymphoid cell accumulation, supported by a network of stromal cells. This network facilitates effective encounter and interaction between antigen-presenting cells and lymphocytes, maximizing effectiveness of the immune response to pathogens. Lymph nodes (LN) and spleen are the best-studied SLO. The spleen has two well-defined areas. In the red pulp, macrophage-lined venous sinuses filter damaged erythrocytes from the blood and allow surveillance of blood-borne pathogens and large antigens. The white pulp is a compartmentalized lymphoid area that is specialized in antigen presentation [1]. Within the white pulp, T and B lymphocytes are segregated into specific areas. Around the central arteriole, T cells are located in the periarteriolar lymphoid sheath (PALS or T cell zone), surrounded by the B cell zone (B cell follicles) [2] . Specific chemokines that attract T and B cells to their respective areas maintain correct organization of the white pulp [1]. The marginal zone (MZ) separates the red and white pulp and contains mainly phagocytic macrophages (marginal metallophilic macrophages (MMM)), marginal zone macrophages (MZ M), marginal zone B cells (MZ B) and DC [2]. In LN, naïve lymphocytes extravasate from the bloodstream through specialized blood vessels known as high endothelial venules (HEV). B and T cell areas surround HEV; B cell folicles are located in the outer cortex and T cells in the diffuse lymphoid tissue of the inner cortex, also known as paracortex [3].

Stromal cells maintain the microarchitectural organization of SLO, allowing correct immune cell movement and interaction, necessary for a protective immune response to pathogens. SLO stromal cells are divided into four populations, defined by gp38 (podoplanin) and CD31 expression. gp38^+^CD31^−^ cells (fibroblastic reticular cells; FRC) form a conduit network for antigen transport and support of immune cell migration, gp38^+^CD31^+^ cells (lymphatic endothelial cells; LEC) build lymph vessels, gp38^−^CD31^+^ cells (blood endothelial cells; BEC) construct cortical vessels and capillaries, including HEV in LN, and gp38^−^CD31^−^ cells (double-negative stromal cells; DN) are a bulk population that includes follicular dendritic cells (FDC) and extrathymic Aire-expressing cells [3], [4]. These four populations are well characterized in LN; FRC, FDC, and BEC are also detected in spleen, where they are likely to have similar characteristics [5]. In mouse spleen, gp38^+^CD31^+^ LEC are reported to form lymphatic vessels [6] that originate around central arteries in the white pulp, join other deep lymphatic vessels that drain into trabeculae, and exit from the spleen hilum [7]. LEC in spleen lymphatic vessels are thought to participate in T cell migration, since lymphocytes within these vessels are CD3^+^ [7]. FRC and FDC secrete cytokines and chemokines and express adhesion molecules that modulate immune cell migration, homeostasis and survival [8], [9], [10].

In SLO, B/T lymphocyte localization and subsequent segregation depend on chemokines secreted by non-hematopoietic stromal cells [3], [4]. In homeostasis, primary B cell follicles contain FDC, which participate in B cell compartment organization and in antigen presentation to B cells. The FDC recruit B cells by secreting CXCL13, which binds to CXCR5 on B cells [11]. The FRC subset forms a network that structures the T cell area [12], [13]; FRC secrete CCL19 and CCL21, chemokines that attract CCR7-expressing T cells and DC to facilitate antigen encounter [8], [14], [15]. FRC constitute the conduit system that allows small antigens and chemokines to migrate to SLO B and T cell areas. Large antigens are excluded from this conduit and are trapped by APC in the spleen MZ or the LN subcapsular sinus. This system extends mainly through the T cell area and also reaches B cell follicles, although less densely [16]. CCL19 and CCL21 are also expressed by BEC and LEC [17].

Members of the TNF family of cytokines have a central role in lymphoid organ development and organization. Lymphotoxin-α (LTα), lymphotoxin-β (LTβ) and tumor necrosis factor (TNF) have varying levels of importance in the development of most SLO [18]. Although lymphotoxin signaling is not necessary for spleen generation, it is needed for red and white pulp segregation, for functional development of spleen white pulp [13], and for appropriate homing and maintenance of B/T segregation [19]. The LT receptor (LTβR) is expressed mainly by irradiation-resistant stromal cells; triggering of LTβR on these cells induces CXCL13 expression in B cell areas and CCL19 and CCL21 in T cell areas, via activation of the “non-canonical” IKKα/NIK-dependent NFκB pathway [20]. LT-deficient mice have disorganized T cell zones; these defects are more severe in spleens of LTα- and LTβR-deficient than LTβ-deficient mice [19]. Impaired signaling through LTβR reduces spleen CXCL13, CCL19 and CCL21 levels, leading to disorganization of white pulp areas [21]. LTα also contributes to lymphangiogenesis [22].

p110δ is a catalytic subunit of class I_A_ PI3K, together with p110α and p110β. It shares a catalytic domain with the other PI3K and binds to a regulatory subunit (p85α or β, p55α, p50α or p50γ). p110δ is expressed preferentially in leukocytes, whereas p110α and p110β are ubiquitous [23]; p110δ is also expressed in neurons [24], in some cancer cell lines [25], [26], and in endothelial cell lines [26], [27], [28]. p110δ has a central role in immune cell processes, including differentiation, activation and development of B and T cells [29], [30], [31], [32], [33], regulatory T cells [34], macrophages [35] and mast cells [36]. p110δ is also essential for generation of immune responses, both primary and secondary (memory) [37], [38]. Analysis of spleen sections shows a severe reduction in MZ B cells in p110δ-deficient mice [31]. Lack of p110δ or its kinase activity greatly impairs germinal center (GC) formation in the spleen after immunization; when these GC form, their size and structure are atypical [30], [31], [32], [39]. These defects in cell segregation and organization in p110δ-deficient mouse SLO suggests that p110δ is expressed in non-hematopoietic stromal cells and that it contributes to the maintenance of cell segregation and organization.

Given the lack of data on p110δ in SLO stromal cells, and on its role in homing and maintenance of B/T segregation, we studied p110δ expression and function in murine spleen and LN. We found p110δ is expressed in gp38^−^CD31^+^ and gp38^+^CD31^+^ spleen stromal cell subpopulations, where it regulates LTβR expression as well as CCL19 and CCL21 production; this suggests a role for p110δ in the control of T cell migration to appropriate spleen areas through the regulation of homeostatic chemokine production by stromal cells.

## Methods

### Mice

p110δ^WT/WT^ and p110δ^D910A/D910A^ mice [30] were bred and maintained in specific pathogen-free conditions in our animal facility; the CNB Ethics Committee for Animal Experimentation approved all animal studies (refs 12021, 12022), in compliance with national and European Union legislation. All efforts were made to minimize suffering.

### Bone marrow reconstitution assays

p110δ^WT/WT^ and p110δ^D910A/D910A^ mice were lethally γ-irradiated (single dose, 10 Gy). After 3–4 h, mice were reconstituted by intravenous injection (tail vein) of total bone marrow from p110δ^WT/WT^ mice. Six weeks after reconstitution, mice were sacrificed, and spleen and LN collected. Half were frozen for immunofluorescence studies, and the remainder used to prepare single-cell suspensions for populations counts and flow cytometry analysis.

### Immune response induction with heat-inactivated *Candida albicans*

Heat-inactivated *Candida albicans* cells (10^6^) were injected into p110δ^WT/WT^, p110δ^D910A/D910A^, reconstituted p110δ^WT/WT^ and p110δ^D910A/D910A^ mice (see Supplement S1 for details). Mice were sacrificed 5 days post-injection, and spleen and LN collected. Half were frozen for immunofluorescence studies, and the remainder used to prepare single-cell suspensions for populations counts and flow cytometry analysis (see Supplement S1).

### Immunofluorescence of SLO sections

Frozen sections of spleen and LN from p110δ^WT/WT^, p110δ^D910A/D910A^, reconstituted p110δ^WT/WT^ and reconstituted p110δ^D910A/D910A^ mice were analyzed by immunofluorescence staining to study distribution and location of immune cell (Thy1.2^+^ and CD3^+^ T cells, MOMA^+^ MMM, B220^+^ B cells, CD11c^+^ DC, see Supplement S1).

### Hematoxylin-eosin staining of spleen sections

Frozen spleen sections from p110δ^WT/WT^, p110δ^D910A/D910A^, reconstituted p110δ^WT/WT^ and reconstituted p110δ^D910A/D910A^ mice were hematoxylin/eosin stained to analyze lymphoid follicle area (see Supplement S1).

### Flow cytometry analysis of immune cell populations

Secondary lymphoid organ cells from p110δ^WT/WT^, p110δ^D910A/D910A^, reconstituted p110δ^WT/WT^ and p110δ^D910A/D910A^ mice were processed and stained for flow cytometry analysis (see Supplement S1).

### Flow cytometry analysis of spleen stromal cells

Stromal cells were extracted using an established protocol [40]. Briefly, mouse spleens were removed, pierced with fine forceps, and placed in ice-cold RPMI-1640 (5 min, on ice). Spleens were dissected, RPMI-1640 removed, and replaced with 2 ml of a fresh enzyme mix composed of dispase (0.8 mg/ml; Gibco) and collagenase IV (0.2 mg/ml; Roche). Tubes were incubated (37°C, 20 min), the cell suspension removed and placed in a fresh tube with ice-cold FACS buffer (3% FBS, 2 mM EDTA in PBS). The remaining spleen was re-incubated with 2 ml fresh enzyme mix (37°C, 10 min), after which the cell suspension was removed and added to fresh tube above. The remaining spleen was reincubated (37°C, 15 min) in 2 ml fresh enzyme mix with vigorous pipetting every 5 min, the cell suspension was removed, placed in the same tube, whose contents were then filtered through a 100 µm nylon mesh. Cells were counted and viability assayed using trypan blue. Cells were stained with CD45 (30-F11, Biolegend), TER119 (TER119, eBioscience), gp38 (8.1.1, eBioscience) and CD31 (MEC 13.3, BD Biosciences) in 100 µl (30 min, 4°C) before analysis on a Cytomix (Beckman Coulter).

### Stromal cell enrichment and cell sorting

Stromal cells were harvested as above. After spleens were fully digested, cells were centrifuged, counted, and the single cell suspension depleted of non-hematopoietic stromal cells using CD45 microbeads in the autoMACS system (Miltenyi) and incubated (20 min, 4°C). CD45-labeled cells were depleted using the autoMACS Depletes program. Purified stromal cells were counted and stained before sorting on a FACSAria III (BD Biosciences).

### qRT-PCR analysis of gene expression

Total RNA was extracted from spleen, LN, and sorted cell populations isolated from p110δ^WT/WT^ and p110δ^D910A/D910A^ mouse spleen. qRT-PCR was performed using specific primers for p110δ, CCL19, CCL21, LTα, LTβ and LTβR (see Supplement S1).

### Statistics

Data are represented as mean ± SD. Most analyses were performed using Student's *t*-test to compare distinct parameters in two independent mouse groups (p110δ^WT/WT^ and p110δ^D910A/D910A^). Where indicated, the Kolmogorov-Smirnov test was used to analyze samples whose distribution is not Gaussian. In all cases, differences were considered significant for p<0.05 (*p<0.05, **p<0.01, ***p<0.001).

## Results

### Analysis of SLO after bone marrow reconstitution assays in homeostatic conditions

**Figure 1:**
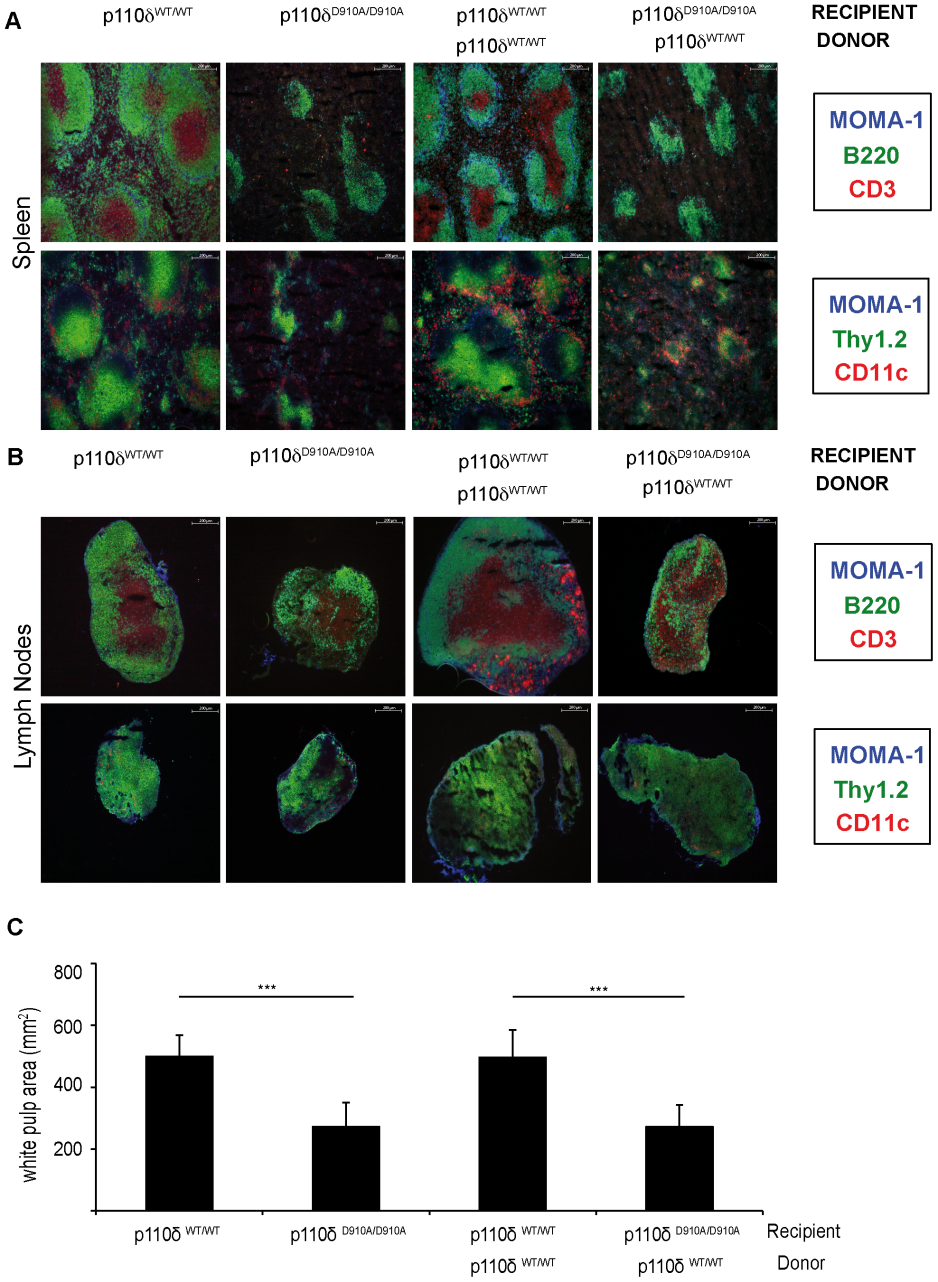
Immunofluorescence analysis of immune cell distribution and white pulp area. Frozen sections of spleen and LN from p110δ^WT/WT^, p110δ^D910A/D910A^, and reconstituted mice were immunofluorescence-stained to detect T cells (CD3^+^, Thy1.2^+^), B cells (B220^+^), MMM (MOMA^+^) and DC (CD11c^+^). Representative images of spleen (**A**) and LN (**B**) sections for all conditions are shown (*n* = 6 mice/condition). Bar = 200 µm. (**C**) Measurement of white pulp area in hematoxylin/eosin-stained frozen spleen sections (3 sections/mouse, 6 mice/condition), quantified with ImageJ software. Mean ± SD; Kolmogorov-Smirnov test, ***p<0.001.

**Figure 2:**
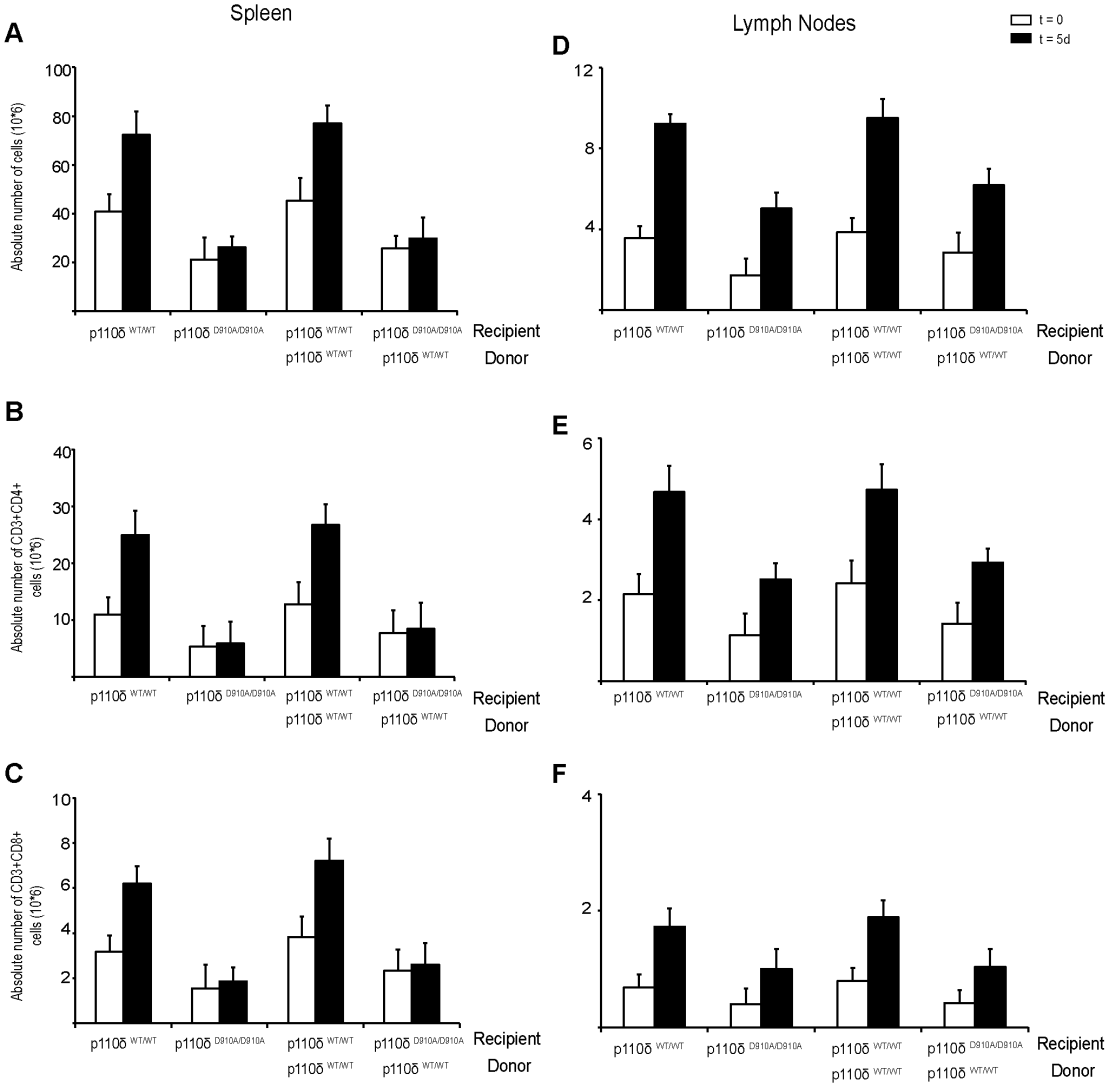
Absolute numbers of spleen and LN total cells, CD4^+^ and CD8^+^ T cells before and after antigen stimulation. Spleens and LN were extracted from p110δ^WT/WT^, p110δ^D910A/D910A^, and reconstituted mice in homeostatic conditions (t = 0) and after antigen stimulation (five days post-injection of inactivated *C. albicans*, t = 5 d). Whole organ cell suspensions were counted to determine total cell number (**A, D**) and stained to determine CD4^+^ T (**B, E**) and CD8^+^ (**C, F**) cell numbers by flow cytometry (*n* = 6 mice/condition). Mean ± SD.

To determine whether defects in the MZ and in MZ B cells in p110δ^D910A/D910A^ mouse spleen ([30], Figure S1, Supplemet S1) were due solely to anomalies in p110δ^D910A/D910A^ hematopoietic cell populations or also to non-hematopoietic stromal cell defects, we used bone marrow reconstitution assays in p110δ^WT/WT^ and p110δ^D910A/D910A^ mice and analyzed SLO in homeostatic conditions. Lethally irradiated p110δ^WT/WT^ and p110δ^D910A/D910A^ mice were reconstituted with total bone marrow from p110δ^WT/WT^ donors. Six weeks after reconstitution, mice were sacrificed for immunofluorescent staining of spleen and LN sections to detect immune cell populations (Figure 1); we also analyzed total cell numbers and lymphoid cell populations of spleen and LN by flow cytometry (Figure 2).

T cell staining of spleen sections showed fewer T cells and more diffuse T cell areas in p110δ^D910A/D910A^ and reconstituted p110δ^D910A/D910A^ recipient mice than in p110δ^WT/WT^ or p110δ^WT/WT^ reconstituted mice (Figure 1A). The defects in the T cell area were less evident in LN sections, although LN were consistently slightly smaller in p110δ^D910A/D910A^ and reconstituted p110δ^D910A/D910A^ recipients than in p110δ^WT/WT^ or p110δ^WT/WT^ reconstituted mice (Figure 1B). Analysis of lymphoid cell distribution in spleen and LN showed that T cell, B cell, MMM, and DC patterns in reconstituted p110δ^WT/WT^ mice resembled those of p110δ^WT/WT^ mice; in reconstituted p110δ^D910A/D910A^ mice, spleen and LN cell distribution was similar to that of p110δ^D910A/D910A^ mice (Figure 1A, spleen; Figure 1B, LN). The pattern was similar when spleen white pulp area was measured; the reconstituted mouse phenotype was thus comparable to that of the recipients (Figure 1C). This result suggested that the effect of stromal cell subsets on immune cell distribution and localization is p110δ activity-dependent.

### SLO analysis after bone marrow reconstitution and antigen stimulation

To test whether p110δ^D910A/D910A^ mouse SLO structural defects in homeostasis are corrected after antigen stimulation, we performed similar studies in bone marrow-reconstituted mice. We studied spleen and LN immune responses simultaneously using heat-inactivated *C. albicans*, which generates concurrent local and systemic immune responses ([41], [42], Figure S2). We injected heat-inactivated *C. albicans* into mice 6 weeks after reconstitution, and sacrificed mice after five days (Figure S2, Supplement S1).

We analyzed total, CD3^+^CD4^+^, and CD3^+^CD8^+^ cell number in p110δ^WT/WT^, p110δ^D910A/D910A^, and bone marrow-reconstituted mouse spleens in homeostasis and after antigen stimulation (Figure 2A–C). After stimulation, total cell numbers increased in spleens from p110δ^WT/WT^ but not from p110δ^D910A/D910A^ mice (Figure 2A). CD4^+^ and CD8^+^ T cell numbers increased similarly in p110δ^WT/WT^ mouse spleen after stimulation, but not in p110δ^D910A/D910A^ mouse spleen (Figure 2B, C), suggesting defective T cell expansion in p110δ^D910A/D910A^ mice. Total spleen cell, CD4^+^ and CD8^+^ T cell numbers increased after stimulation compared to homeostatic conditions in reconstituted p110δ^WT/WT^, but not in p110δ^D910A/D910A^ recipient mice (Figure 2A–C), indicating that spleen stromal cells in p110δ^D910A/D910A^ mice might not contribute appropriately to T cell expansion in response to heat-inactivated *C. albicans. W*e analyzed total, CD3^+^CD4^+^ and CD3^+^CD8^+^ cell number in p110δ^WT/WT^, p110δ^D910A/D910A^, and bone marrow-reconstituted mouse LN in homeostasis and after antigen stimulation (Figure 2D–F). LN from p110δ^WT/WT^ and p110δ^D910A/D910A^ mice showed an increase in total cell number, which was smaller in p110δ^D910A/D910A^ than in p110δ^WT/WT^ mice (Figure 2D). A similar increase was observed for CD4^+^ and CD8^+^ T cells in LN (Fig. 2E, F), indicating that p110δ^WT/WT^ and p110δ^D910A/D910A^ mouse LN respond to *C. albicans* stimulation, although the response was slightly lower in p110δ^D910A/D910A^ than in p110δ^WT/WT^ mice. After mouse reconstitution, total LN cell numbers increased after antigenic stimulation in p110δ^WT/WT^, and to a lesser extent in p110δ^D910A/D910A^ recipients (Figure 2D). Results were similar for LN CD4^+^ and CD8^+^ T cells, suggesting that LN stroma supports the T cell immune response to heat-inactivated *C. albicans*.

**Figure 3:**
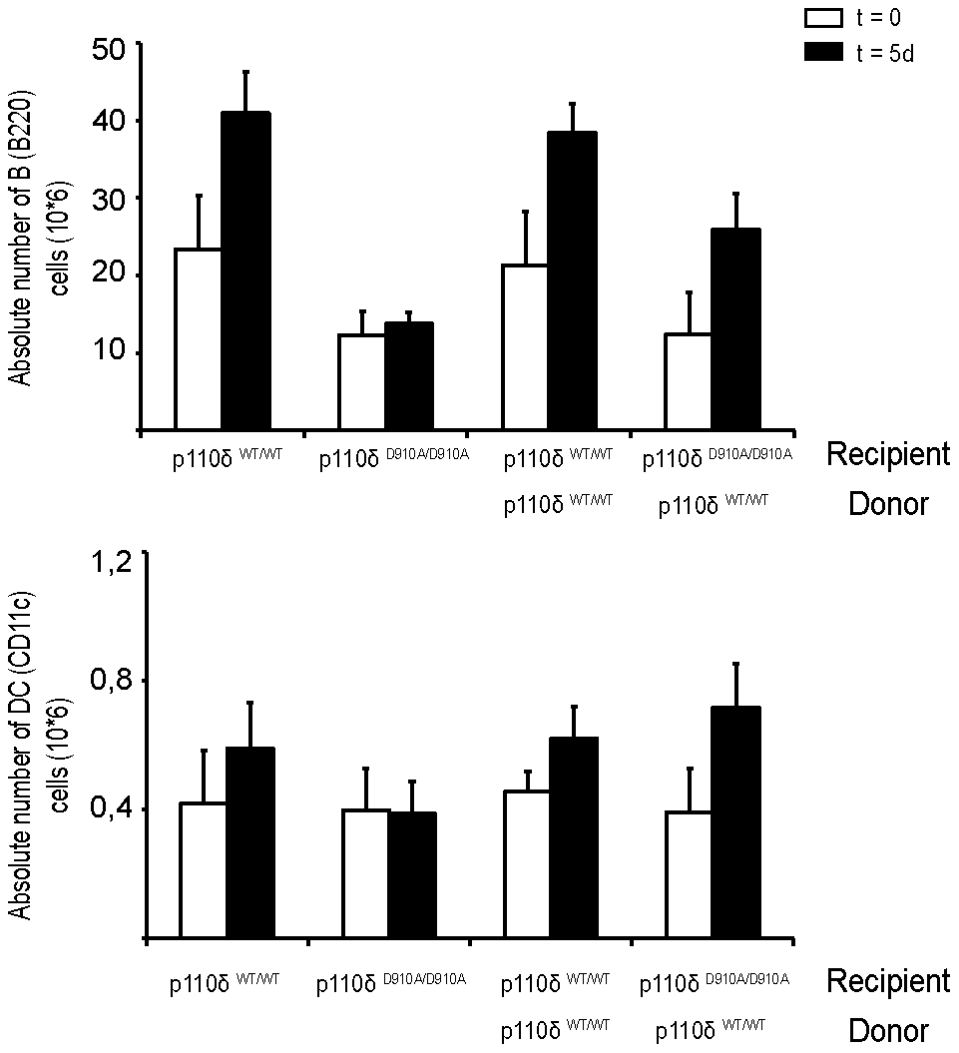
Absolute numbers of spleen B cells and DC before and after antigen stimulation. Spleens were extracted from p110δ^WT/WT^, p110δ^D910A/D910A^, and reconstituted mice in homeostatic conditions (t = 0) and after antigen stimulation (five days post-injection of inactivated *C. albicans*, t = 5 d). B cell (**A**) and DC (**B**) were stained and cell numbers determined by flow cytometry (*n* = 6 mice/condition). Mean ± SD.

To determine whether other spleen cell types involved in the immune response to heat-inactivated *C. albicans* were affected, we analyzed B cell (B220^+^) and dendritic cell (DC, CD11c^+^) numbers in p110δ^WT/WT^, p110δ^D910A/D910A^, and bone marrow-reconstituted mouse spleens in homeostasis and after antigen stimulation (Figure 3A, B). B cell numbers were increased in p110δ^WT/WT^ but not in p110δ^D910A/D910A^ mouse spleen (Figure 3A). DC cell numbers showed a similar increase in p110δ^WT/WT^ spleen after stimulation, but not in spleens from p110δ^D910A/D910A^ mice (Figure 3B), suggesting defective B cell and DC expansion in p110δ^D910A/D910A^ spleens. B cell and DC numbers increased after antigen stimulation compared to homeostatic conditions in reconstituted p110δ^WT/WT^ and p110δ^D910A/D910A^ recipient mice (Figure 3A, B). These results suggest that spleen stromal cells lacking p110δ activity contributed to correct B cell and DC expansion in response to heat-inactivated *C. albicans*. The defect in spleen B cell and DC expansion in p110δ^D910A/D910A^ mice after antigen stimulation is probably due to the role of p110δ in the function of these cell types [30], [31], [32], [43].

### FACS analysis of spleen stromal cell populations in p110δ^WT/WT^ and p110δ^D910A/D910A^ mice

**Figure 4:**
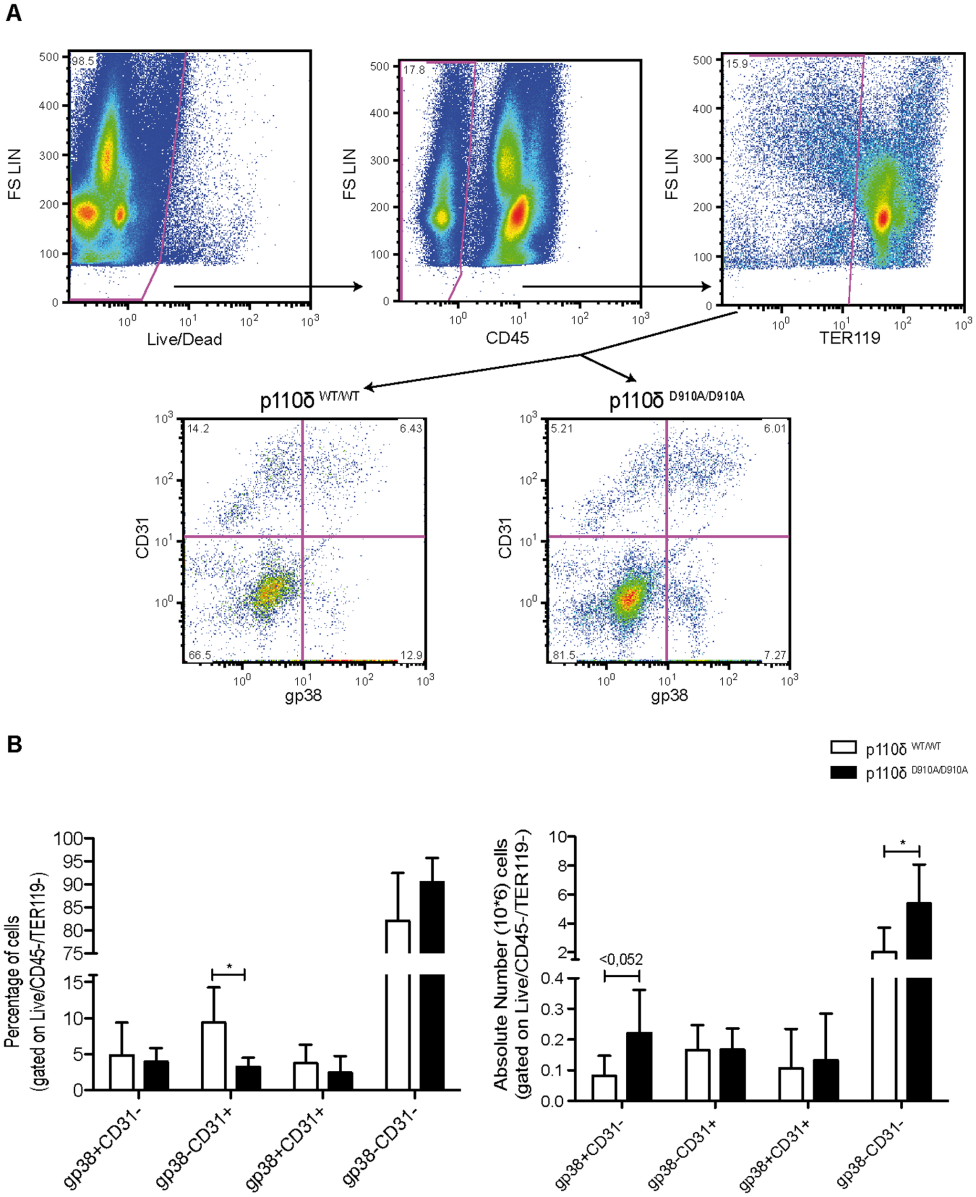
FACS analysis of stromal cell populations in spleen from p110δ^WT/WT^ and p110δ^D910A/D910A^ mice. Spleens from p110δ^WT/WT^ and p110δ^D910A/D910A^ mice were processed and stained with anti-CD45, -TER119, -CD31, and -gp38 mAb. A) Representative gating strategy for the analysis of stromal cell populations. Stromal cells were gated via the exclusion of dead, CD45-, and TER119-positive cells. B) Quantification of the percentage and absolute number of stromal cell populations in spleens of p110δ^WT/WT^ and p110δ^D910A/D910A^ mice (*n* = 3 experiments/spleen, 6 mice/group). Student's *t*-test, *p<0.05.

To evaluate the effect of lack of p110δ activity on the percentages and numbers of the four stromal cell subsets defined by gp38 and CD31 in spleen (FRC, LEC, BEC, DN), we used FACS to analyze p110δ^WT/WT^ and p110δ^D910A/D910A^ mouse spleen cells (Figure 4A). Analysis of CD45^−^TER119^−^ spleen cells showed a significant decrease in the percentage of gp38^−^CD31^+^ cells (BEC) in p110δ^D910A/D910A^ compared to p110δ^WT/WT^ mice (Figure 4A). We also found an increase in total number of gp38^+^CD31^−^ (FRC) and gp38^−^CD31^−^ (DN) cells in p110δ^D910A/D910A^ compared to p110δ^WT/WT^ mice (Figure 4B).

### p110δ mRNA expression in spleen stromal cell populations

**Figure 5:**
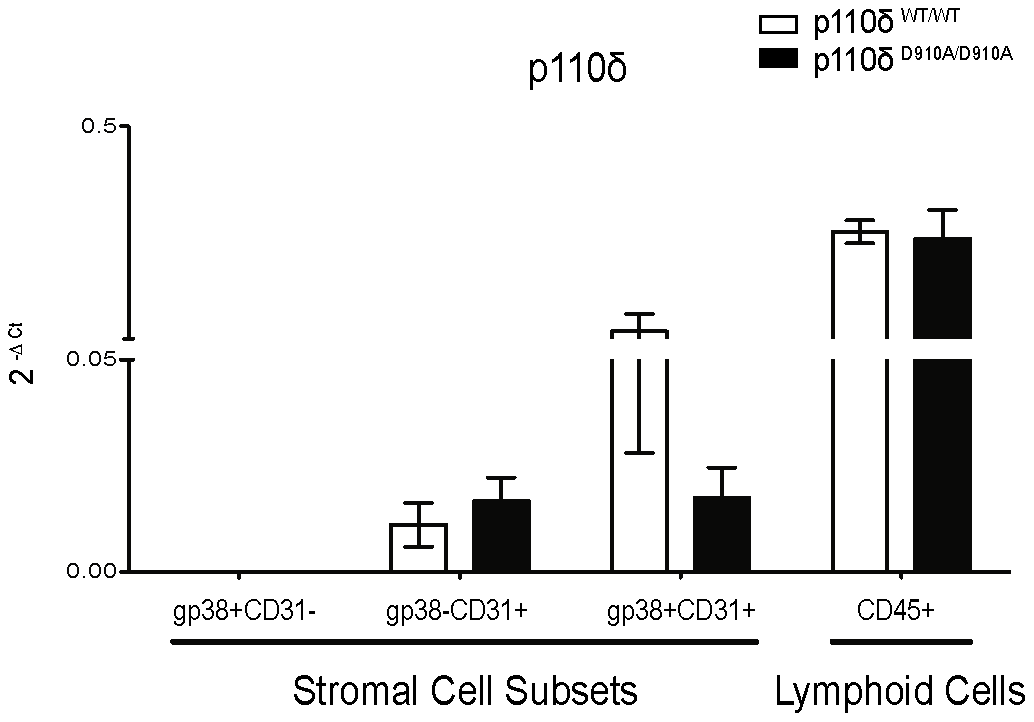
p110δ mRNA expression in spleen stromal cell populations from p110δ^WT/WT^ and p110δ^D910A/D910A^ mice. Total RNA was extracted from sorted p110δ^WT/WT^ and p110δ^D910A/D910A^ spleen stromal cell subsets (*n* = 5 mice/genotype). Lymphoid cells (CD45^+^) were sorted as control. Expression of p110δ mRNA was analyzed by qRT-PCR. Normalized quantities (mean 2^−ΔCt^) of p110δ mRNA are shown.

To test whether p110δ mRNA was expressed in spleen stroma cells, the four stromal cell subsets defined by gp38/CD31 expression were sorted from p110δ^WT/WT^ and p110δ^D910A/D910A^ mouse spleens and p110δ expression analyzed by RT-PCR. As a positive control, CD45^+^ (lymphoid) cells were also sorted. Although lymphoid cells express higher p110δ mRNA levels, gp38^+^CD31^+^ cells (LEC) and to a lesser extent, gp38^−^CD31^+^ cells (BEC) also expressed p110δ mRNA, whereas gp38^+^CD31^−^ (FRC) cells did not (Figure 5). Within the LEC population, p110δ mRNA levels were notably reduced in p110δ^D910A/D910A^, whereas they were similar in BEC and lymphoid cells (Figure 5).

### qRT-PCR of homeostatic chemokines and TNF family members in spleen, LN and spleen stromal cell subsets in p110δ^WT/WT^ and p110δ^D910A/D910A^ mice

**Figure 6:**
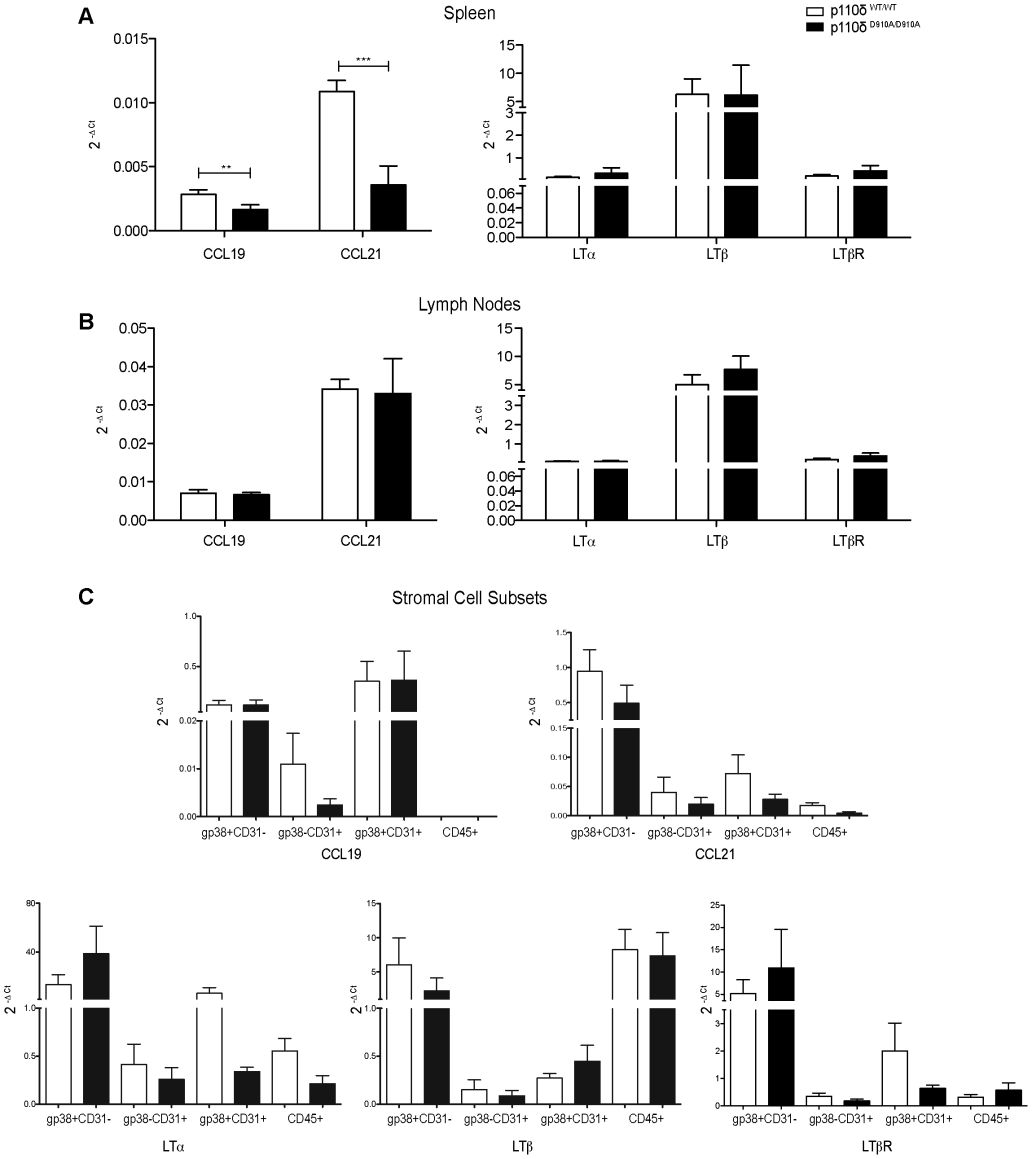
qRT-PCR analysis of homeostatic chemokines and TNF family members in spleen, LN and spleen stromal cell subsets from p110δ^WT/WT^ and p110δ^D910A/D910A^ mice. Total RNA was extracted from p110δ^WT/WT^ and p110δ^D910A/D910A^ spleen, LN, and sorted spleen stromal cell subsets (*n* = 5 mice/genotype). Expression of CCL19, CCL21, LTα, LTβ and LTβR was analyzed by qRT-PCR in spleen (**A**), LN (**B**), and stromal cell subsets (**C**). Normalized quantities (mean 2^−ΔCt^) of mRNA are depicted. Student's *t*-test, *p<0.05, **p<0.01, ***p<0.001.

T lymphocyte homing and retention in SLO depends on secretion of the homeostatic chemokines CCL19, CCL21 and CXCL13 by non-hematopoietic stromal cells. LTα, LTβ, and TNF trigger stromal cell production of these homeostatic chemokines. We used qRT-PCR to analyze the expression of CCL19 and CCL21 and of TNF family proteins (LTα, LTβ, LTβ-receptor) in total RNA extracts of whole spleens and LN from p110δ^WT/WT^ and p110δ^D910A/D910A^ mice. Expression of CCL21 and to a lesser extent, that of CCL19 were lower in total RNA extracts from p110δ^D910A/D910A^ than from p110δ^WT/WT^ mouse spleens (Figure 6A); there were no differences in LN from either genotype (Figure 6B). Analysis of mRNA levels of TNF family proteins or their receptor LTβR showed no differences in spleen or LN (Figure 6A, B).

To better define the defects identified in whole spleen extracts, we separated the spleen gp38/CD31-defined stromal cell subsets by cell sorting and analyzed chemokine and TNF family mRNA expression in extracts of each population. Analysis showed a reduction in CCL19 mRNA levels only in p110δ^D910A/D910A^ gp38^−^CD31^+^ (BEC) compared to p110δ^WT/WT^; gp38^+^CD31^−^ (FRC) and gp38^+^CD31^+^ (LEC) subsets expressed the highest levels (Figure 6C). CCL21 mRNA levels were slightly reduced in all spleen stromal populations, with the highest levels in gp38^+^CD31^−^ (FRC, Figure 6C). These chemokines were barely detectable in lymphoid cells (Figure 6C).

For TNF family proteins, the gp38^+^CD31^−^ (FCR) p110δ^D910A/D910A^ population expressed the highest LTα levels, whereas p110δ^D910A/D910A^ gp38^+^CD31^+^ (LEC) showed a significant reduction compared with p110δ^WT/WT^. LTβ was produced mainly by lymphoid cells and by gp38^+^CD31^−^ (FRC), and p110δ^WT/WT^ and p110δ^D910A/D910A^ populations showed no notable differences. LTβR was expressed mainly by gp38^+^CD31^−^ (FRC) and gp38^+^CD31^+^ (LEC) in p110δ^WT/WT^, with greatly reduced expression in p110δ^D910A/D910A^ gp38^+^CD31^+^ (LEC) (Figure 6C).

## Discussion

The immune response is controlled by lymphoid and stromal cell function and location in SLO [4]. The PI3K p110δ isoform is expressed preferentially by leukocytes, although it is also detected in other cell types [24], [25], [26], [27], [28]. MZ B cell numbers are extremely low in p110δ-deficient mouse spleen [31], and lack of p110δ or its kinase activity severely impairs germinal center (GC) formation in the spleen after immunization [30], [31], [32], [39]. We tested whether this isoform is expressed in SLO stromal cells, and whether expression mediates cell location and compartimentalization in these organs.

Reconstitution assays have been used to analyze and confirm specific p110δ functions in memory T cells; lethally irradiated WT mice were reconstituted with purified memory T cell subsets (CD62L^hi^ central memory T cells and CD62L^lo^ effector memory T cells) from p110δ^D910A/D910A^ and p110δ^WT/WT^ mice [35]. Using reconstitution assays with total bone marrow from p110δ^WT/WT^ mice, we tested whether stromal cells have a role in SLO reconstitution (p110δ^WT/WT^-reconstituted p110δ^WT/WT^, p110δ^WT/WT^-reconstituted p110δ^D910A/D910A^ mice). Immunohistochemical analysis of p110δ^D910A/D910A^ and reconstituted p110δ^D910A/D910A^ recipient mouse spleen showed reduced T cell staining and more diffuse T cell areas than in p110δ^WT/WT^ or p110δ^WT/WT^ reconstituted mice. In addition, in p110δ^D910A/D910A^ mice reconstituted with p110δ^WT/WT^ bone marrow, spleen CD4^+^ and CD8^+^ T cell numbers did not increase in response to heat-inactivated *C. albicans*, suggesting that a p110δ^D910A/D910A^ stroma defect impedes a correct immune response. We thus hypothesized a role for p110δ in stromal cell function in the spleen.

SLO stromal cells are divided into four populations as defined by gp38 and CD31 expression, LEC (gp38^+^CD31^+^), FRC (gp38^+^CD31^−^), BEC (gp38^−^CD31^+^), and double negative cells (gp38^−^CD31^−^) [3], [4]. FACS analysis of spleen stromal cell populations showed a significant decrease in the percentage of gp38^−^CD31^+^ cells in p110δ^D910A/D910A^ mice, which paralleled an increase in total gp38^+^CD31^−^ and gp38^−^CD31^−^ cells. This result suggested that p110δ is expressed differently in each spleen stromal population. As there are no reports of p110δ expression in SLO stromal cell subsets, we sorted the four subpopulations from p110δ^WT/WT^ and p110δ^D910A/D910A^ spleen and tested for p110δ mRNA expression by qRT-PCR. In addition to its expression in lymphoid cells, p110δ was detected in spleen LEC and BEC subsets. p110δ mRNA levels in LEC were significantly lower in p110δ^D910A/D910A^ than in p110δ^WT/WT^ spleen.

T homing and compartmentalization in SLO requires chemokine secretion by stromal cells. FRC secrete the homeostatic chemokines CCL19 and CCL21 [3], which are also produced by LEC and BEC [17]. Analysis of their expression in total RNA extracts of p110δ^D910A/D910A^ spleen showed significantly lower levels of CCL21 and, to a lesser extent, of CCL19 than p110δ^WT/WT^ spleen; comparison of p110δ^D910A/D910A^ and p110δ^WT/WT^ LN showed no differences in CCL19 and CCL21 levels. The spleen defects led us to analyze chemokine expression in the four stromal subpopulations. Lack of p110δ catalytic activity significantly impaired CCL19 production by BEC, and reduced CCL21 production in all populations. This CCL19 and CCL21 expression defect in the stromal cells could give rise to the abnormal B/T cell segregation observed in p110δ mouse spleen.

LTα, LTβ and TNF participate to some degree in the development of most SLO [18]. Lymphotoxin signaling is necessary for red and white pulp segregation, as well as for correct B/T cell homing and maintenance of segregation [19]. We found no differences in spleen or LN LTα and LTβ expression between p110δ^WT/WT^ and p110δ^D910A/D910A^ mice. When we analyzed mRNA in specific spleen stromal cell populations, however, expression of LTα and LTβR expression were significantly lower in p110δ^D910A/D910A^ LEC and somewhat less so in BEC compared to those of p110δ^WT/WT^ mice; no differences were observed in LTβ expression. LTα^−/−^, LTβ^−/−^ and LTβR^−/−^ defects differed in SLO [44], [45], [46] [47]. The p110δ^D910A/D910A^ spleen phenotype is similar to that of mice in which LTαβ-LTβR interaction is blocked by a soluble LTβR-IgG1 fusion protein [48], and includes loss of MZ and of T/B cell segregation, although segregation was normal in LN. Low LTβR expression in LEC and BEC appears to be the primary cause of these spleen defects in p110δ^D910A/D910A^ mice, together with low CCL19 and CCL21 production, which affects T/B cell migration and compartmentalization. The need for LTα for B/T cell segregation in spleen white pulp, whereas TNFR-I is necessary for B/T cell segregation in LN [49], is consistent with the lesser defects in p110δ^D910A/D910A^ LN compared with spleen.

In summary, we found p110δ expression by gp38^−^CD31^+^ and gp38^+^CD31^+^ spleen stromal cells. Lack of p110δ activity in these populations correlated with lower LTβR, CCL19 and CCL21 mRNA levels. These findings could explain the lower T cell numbers and more diffuse T cell areas observed in p110δ^D910A/D910A^ mouse spleen, and the lower T cell expansion after antigen stimulation observed in p110δ^D910A/D910A^ compared with p110δ^WT/WT^.

## Supporting Information

Supplement S1 **Supporting Materials and Methods, Results and References.**
(DOC)

Figure S1 **Distribution of immune cell types from p110δ^WT/WT^ and p110δ^D910A/D910A^ spleen marginal zone.** Histological sections from p110δ^WT/WT^ and p110δ^D910A/D910A^ spleens were immunofluorescent stained for marginal zone immune cell types. (**A**) MZB (B220^+^ surrounding MOMA^+^ cells around spleen follicles) and MMM (MOMA^+^) (*n* = 4 mice/genotype). (**B**) MZM (SIGNR1^+^) and MMM (MOMA^+^) (*n* = 4 mice/genotype). Bar = 200 µm.
(TIF)

Figure S2 **Immune response in p110δ^WT/WT^ mice injected with heat-inactivated** ***C. albicans*** **.** p110δ^WT/WT^ mice received i.p. injections of heat-inactivated *C. albicans* for the indicated times (0, 2, 5, 7, 9 and 21 d) to stimulate an immune response. Total CD4^+^ T cells from p110δ^WT/WT^ spleens (**A**) and LN (**B**) were counted before (t = 0) and several times after *C. albicans* injection (*n* = 6–10 mice). Mean ± SD.
(TIF)
