# Supplementary material for: PI3K p110δ Is Expressed by gp38−CD31+ and gp38+CD31+ Spleen Stromal Cells and Regulates Their CCL19, CCL21, and LTβR mRNA Levels
Source: PLoS One. 2013 Aug 29;8(8):e72960. doi: 10.1371/journal.pone.0072960 (PMC3757018; doi:10.1371/journal.pone.0072960)
Supplement: Supplement S1 — Supporting Materials and Methods, Results and References. (DOC) [file pone.0072960.s001.doc]

**SUPPLEMENT S1**

**Supporting Materials and Methods**

**Hematoxylin-eosin staining of frozen spleen sections**

Serial sections of spleens from p110δ^WT/WT^, p110δ^D910A/D910A^, reconstituted p110δ^WT/WT^ and reconstituted p110δ^D910A/D910A^ mice were defrosted (RT, 45 min), fixed in 4% paraformaldehyde (PFA) (Sigma-Aldrich, St. Louis, MO; 10 min), washed in distilled water (3 x 3 min) and in running water (3 min), and incubated in Mayer’s hematoxylin (Sigma; 15 min) and washed in water (10 min). Sections were incubated in 1% eosin (2-4 min), washed twice in 70% ethanol, dehydrated in 95% ethanol (3 min), 100% ethanol (3 min) and xylene (3 min), slides were mounted in Entellan (Merck) and dried at 37ºC. Images were acquired using a Leica microscope (vertical Leitz DM RB) with an adapted Olympus (DP70) camera.

**Immunofluorescence of SLO sections**

Frozen sections from p110δ^WT/WT^, p110δ^D910A/D910A^, reconstituted p110δ^WT/WT^ and reconstituted p110δ^D910A/D910A^ mice spleen and paraaortic lymph nodes (LN) were stored at ‑80ºC. Before staining, slides were thawed (RT, 45 min) until sections were completely dry, then fixed in 4% PFA (RT, 10 min) and washed in PBS. When using biotinylated antibodies, sections were pretreated with the Avidin/Biotin Blocking kit (Vector Laboratories, Burlingame, CA), slides were incubated in blocking solution ((1% bovine serum albumin (BSA; Sigma) + 10% goat serum in PBS; RT, 30 min)) and incubated without washing (4ºC, overnight (o/n)) with primary antibody rat anti-mouse CD45R/B220, purified hamster anti-mouse CD3 for IHQ, rat anti-mouse CD90.2/Thy1.2 (all from BD Pharmingen, San Diego, CA), monoclonal anti-mouse metallophilic macrophages/MOMA-biotin (Acris Antibodies, Herford, Germany), or purified hamster anti-mouse CD11c (Caltag Labs, Buckingham, UK). Sections were washed with PBS and incubated (RT, 45 min) with the appropriate secondary antibody (streptavidin-Cy5, goat anti-rat-Alexa488, goat anti-hamster IgG-Cy3 (all from Molecular Probes/Invitrogen, Carlsbad, CA)). Slides were washed again and mounted in Fluoromount‑G (Southern Biotech, Birmingham, AL).

***Candida albicans* growth and inactivation**

*Candida albicans,* obtained from the National Collection of Pathogenic Fungi (NCPF-3153, serotype A), is a nonpathogenic strain (ATCC 36801; Biosafety level 1). It was cultured in YPD yeast medium (0.2% bacto yeast extract, 0.1% bacto peptone (BD Biosciences, Franklin Lakes, NJ) in distilled water) supplemented with 2 M glucose and ampicillin (1/1000). The sample was diluted several times in liquid YPD medium and cultured in YPD agar (0.2% bacto yeast extract + 0.1% bacto peptone + 0.24% agar (BD Biosciences); 24ºC, 24-48 h). A single colony from one plate was cultured in 500 ml liquid YPD medium (130 rpm, 24ºC, 18 h) to obtain a yeast suspension, which was centrifuged (750 xg, 4ºC, 1 h), the pellet washed in sterile PBS, and centrifuged again (750 xg, 4ºC, 1 h). This *C. albicans* pellet was heat-inactivated by incubation in a water bath (65‑67ºC, 4 h) and tubes shaken every 10-15 min (inactivation was confirmed by lack of *C. albicans* growth after culture in YPD agar (24ºC, 48-72 h)). *C. albicans* aliquots were stored in sterile PBS (4 x 10^8^ cells/ml, 4ºC).

**Immune response induction by injection of heat-inactivated *C. albicans***

To confirm in our p110δ^WT/WT^ mice that heat-inactivated *C. albicans* injection induced a CD4^+^ T cell systemic expansion as reported [1], [2], we injected 10^6^ heat‑inactivated *C. albicans* cells intraperitoneally (i.p.) in p110δ^WT/WT^ mice. We analyzed total CD4^+^ T cell numbers in spleen and para-aortic LN at various times post-inoculation (2, 5, 7, 9 or 21 days) by flow cytometry. We used paraaortic LN, as they are the main LN that drain the peritoneal cavity. We found CD4^+^ T cell expansion in p110δ^WT/WT^ spleen and LN (Fig. S2A, B).

**Flow cytometry analysis of immune cell populations**

Single-cell suspensions were obtained from spleen, peripheral and paraaortic LN. Total cells were counted by Trypan blue dye exclusion. Cells (0.5-1 x 10^6^ cells/well) were plated in 96‑well plates and washed twice in staining PBS (1% fetal calf serum (FCS) + 0.5% BSA + 0.065% NaN_3_ in PBS). Between washes, plates were centrifuged (800 xg, 4ºC, 5 min). Cells were incubated in the dark (4ºC, 20 min) with appropriate anti-CD3-APC-eFluor780, ‑CD8-eFluor450 (both from eBioscience), -CD4-PE, -B220-Pacific Blue, and -CD11c-APC antibodies (all from Beckman Coulter, Indianapolis, IN), then washed in staining PBS and transferred to cytometry tubes for FACS analysis (Gallios Flow Cytometer, Beckman Coulter). Data were analyzed with Kaluza software.

**qRT-PCR analysis of gene expression**

RNA was extracted with TRI Reagent (Sigma) from homogenates of frozen spleen and peripheral LN from p110δ^WT/WT^ and p110δ^D910A/D910A^ mice. cDNA was obtained from 1 μg total RNA using the High Capacity cDNA Reverse Transcription Kit 496 thermocycler (MWG AG Biotech, Ebersberg, Germany). Several dilutions of each cDNA were used as templates for PCR amplification in an ABI PRISM 7900HT (Applied Biosystems, Carlsbad, CA) using HOTFire Pol 5x buffer (Solis Biodyne, Tartu, Estonia). Primers specific for p110δ, CCL19, CCL21, lymphotoxin-α (LTα), lymphotoxin-β (LTβ), or lymphotoxin receptor (LTβR) were used. Amplification was performed at 95ºC, 10 min; 40 cycles, 95ºC, 15 sec; 60ºC, 20 sec; 72ºC, 30 sec. To confirm amplification purity, a dissociation curve was generated at 95ºC, 15 sec; 60ºC, 15 sec and 2% ramp to 95ºC. mRNA levels were quantified using the 2^-ΔCt^ method and normalized to an endogenous reference (β‑actin or GAPDH) and relative to a calibrator. Results were analyzed using SDS v2.2.2.

| **Gene** | **Forward primer (5´- 3´)** | **Reverse primer (5´- 3´)** |
| --- | --- | --- |
| **p110δ** | TaqMan-MGB p110δ (Mm00435674_m1), Applied Biosystems | |
| **CCL19** | TGGTTCTCTGGACCTTCCCA | CACGATGTTCCCAGGGATG |
| **CCL21** | GGGAAACAAAGCCCCGG | GCTGTGTCTGTTCAGTTCTCTTG |
| **LTα** | cacactgccgcttcctctat | ccgagcagtgtcatgtgg |
| **LTβ** | cctggtgaccctgttgttg | tgctcctgagccaatgatct |
| **LTβR** | gctccaggtacctcctactcg | atggccagcagtagcattg |
| **GAPDH** | agaaaccctggaccaccca | ctccctcacaatttccatccc |
| **RNA 18s** | gagaaacggctaccacatcc | gggtcgggagtgggtaat |
| **β-actin** | ggctcctagcaccatgaaga | ccaccgatccacacagagta |

**Supporting Results**

**Immune cell type distribution in the marginal zone of p110δ^WT/WT^ and p110δ^D910A/D910A^ mouse spleen**

Mice lacking p110δ have very few marginal zone (MZ) B cells (MZB) beyond the marginal sinus (delimitated by MZ metallophilic-macrophages (MMM)) [3]. We studied the distribution of various spleen cell types by immunofluorescence staining of frozen spleen sections from p110δ^WT/WT^ and p110δ^D910A/D910A^ mice. Marginal zone cell types were disorganized in p110δ^D910A/D910A^ mice. The main immune cell types whose distribution was affected were MMM and MZB (Fig. S1A and reference [4]), and marginal zone macrophages (MZM) (Fig. S1B).

**Supporting References**

1. Rosati E, Scaringi L, Cornacchione P, Fettucciari K, Sabatini R, et al. (1995) Cytokine response to inactivated Candida albicans in mice. Cell Immunol 162: 256-264.

2. Scaringi L, Rosati E, Cornacchione P, Fettucciari K, Sabatini R, et al. (1995) Local and systemic immune response to inactivated Candida albicans in mice. Nat Immun 14: 234-249.

3. Clayton E, Bardi G, Bell SE, Chantry D, Downes CP, et al. (2002) A crucial role for the p110delta subunit of phosphatidylinositol 3-kinase in B cell development and activation. J Exp Med 196: 753-763.

4. Durand CA, Hartvigsen K, Fogelstrand L, Kim S, Iritani S, et al. (2009) Phosphoinositide 3-kinase p110 delta regulates natural antibody production, marginal zone and B-1 B cell function, and autoantibody responses. J Immunol 183: 5673-5684.
