# Supplementary figures and images for: PI3K p110δ Is Expressed by gp38−CD31+ and gp38+CD31+ Spleen Stromal Cells and Regulates Their CCL19, CCL21, and LTβR mRNA Levels
Source: PLoS One. 2013 Aug 29;8(8):e72960. doi: 10.1371/journal.pone.0072960 (PMC3757018; doi:10.1371/journal.pone.0072960)

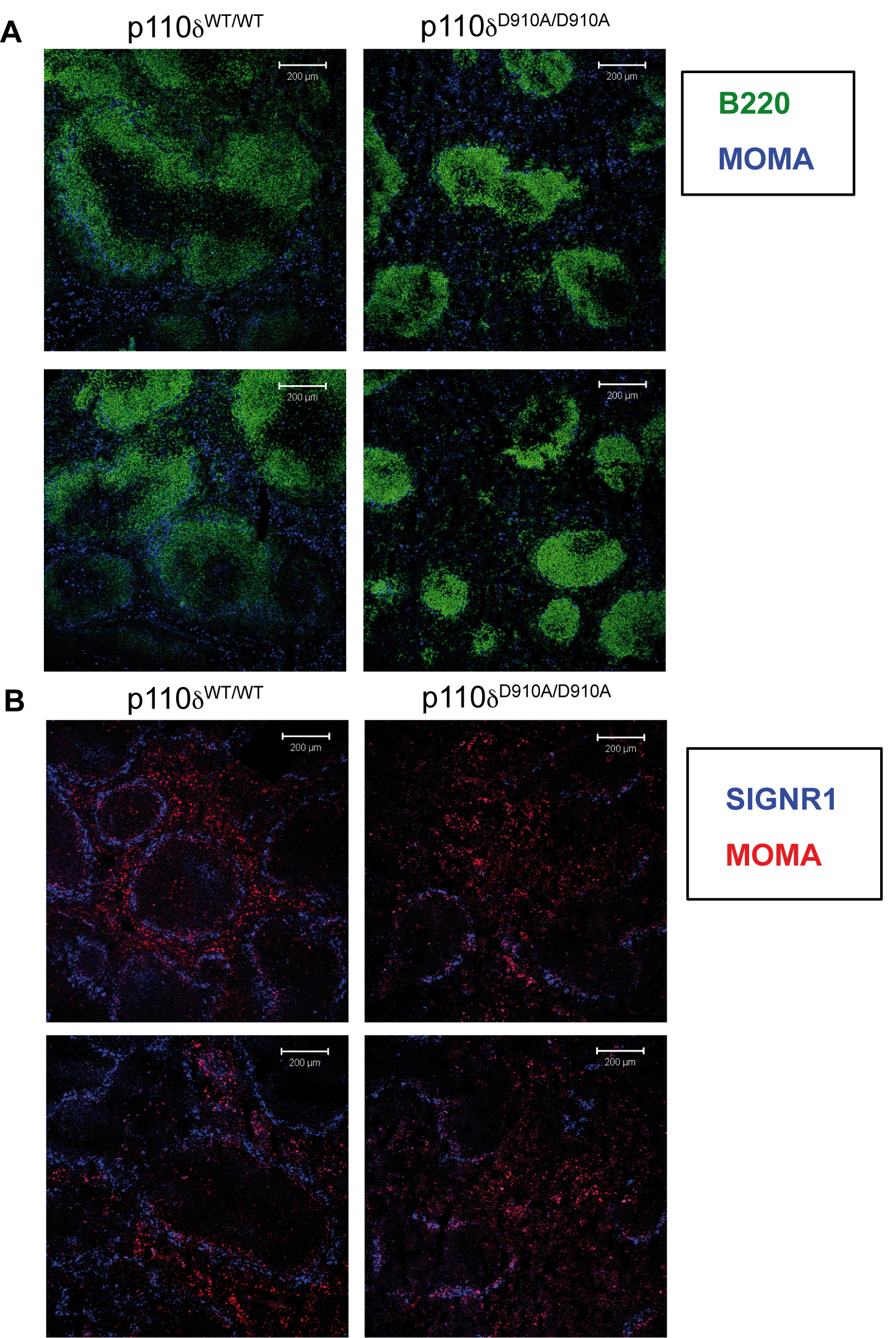

Supplement: Figure S1 — Distribution of immune cell types from p110δWT/WT and p110δD910A/D910A spleen marginal zone. Histological sections from p110δWT/WT and p110δD910A/D910A spleens were immunofluorescent stained for marginal zone immune cell types. (A) MZB (B220+ surrounding MOMA+ cells around spleen follicles) and MMM (MOMA+) (n = 4 mice/genotype). (B) MZM (SIGNR1+) and MMM (MOMA+) (n = 4 mice/genotype). Bar = 200 µm. (TIF) [file pone.0072960.s002.tif]

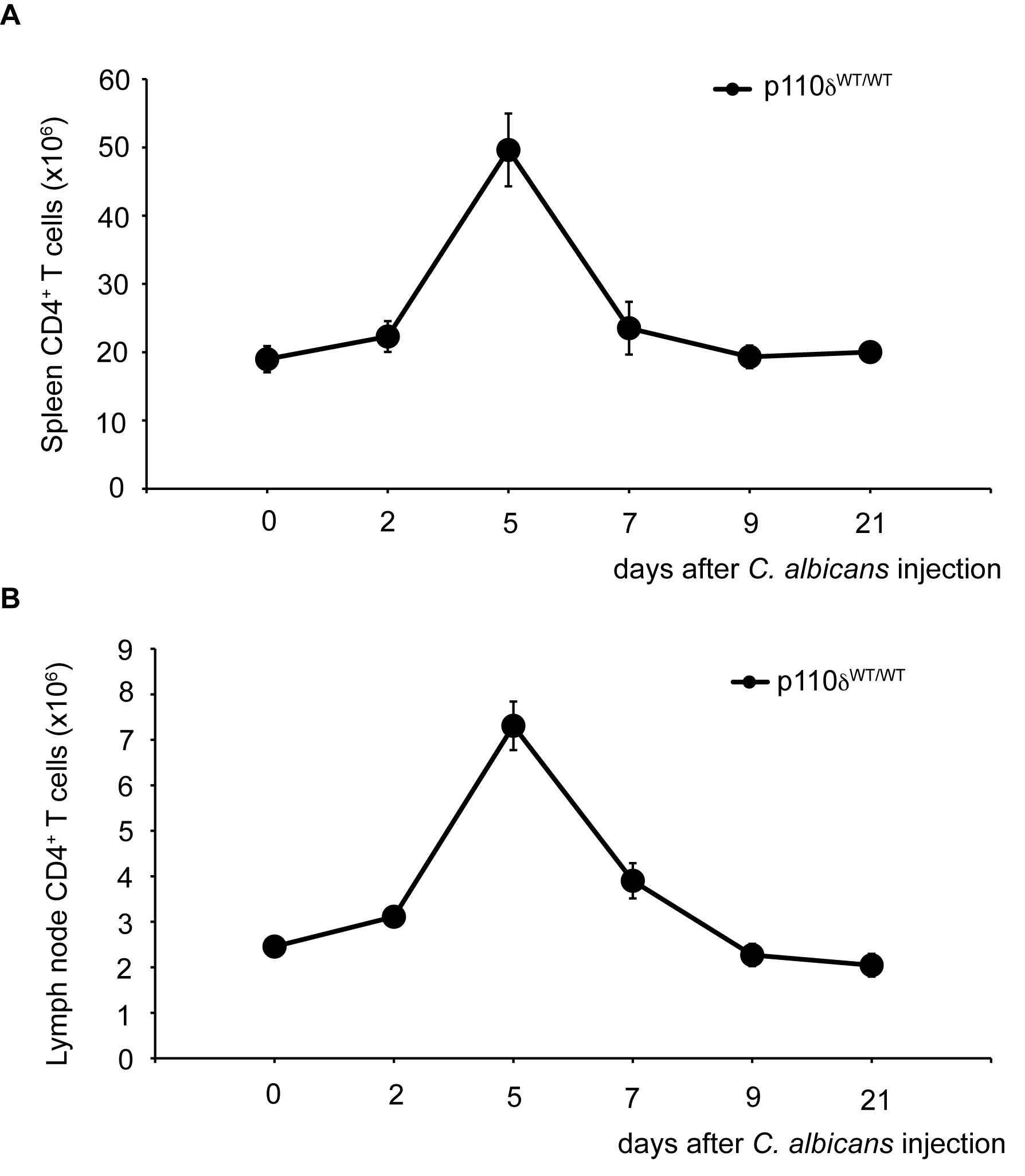

Supplement: Figure S2 — Immune response in p110δWT/WT mice injected with heat-inactivated C. albicans . p110δWT/WT mice received i.p. injections of heat-inactivated C. albicans for the indicated times (0, 2, 5, 7, 9 and 21 d) to stimulate an immune response. Total CD4+ T cells from p110δWT/WT spleens (A) and LN (B) were counted before (t = 0) and several times after C. albicans injection (n = 6–10 mice). Mean ± SD. (TIF) [file pone.0072960.s003.tif]
